# New Insights into Gonorrhea Natural History and Protection by 4CMenB, and a Call for More Analyses of Existing Studies and Enhanced Data Collection

**DOI:** 10.1093/ofid/ofaf214

**Published:** 2025-04-10

**Authors:** Peter J White, Trystan Leng, Dariya Nikitin, Lilith K Whittles

**Affiliations:** MRC Centre for Global Infectious Disease Analysis and NIHR Health Protection Research Unit in Modelling and Health Economics, Imperial College London, London, UK; Modelling and Economics Unit, UK Health Security Agency, London, UK; MRC Centre for Global Infectious Disease Analysis and NIHR Health Protection Research Unit in Modelling and Health Economics, Imperial College London, London, UK; MRC Centre for Global Infectious Disease Analysis and NIHR Health Protection Research Unit in Modelling and Health Economics, Imperial College London, London, UK; MRC Centre for Global Infectious Disease Analysis and NIHR Health Protection Research Unit in Modelling and Health Economics, Imperial College London, London, UK

To the  Editor—The 4CMenB meningitis B vaccine has been reported by multiple studies to provide partial protection against gonorrhea, with trial-based evidence currently inconclusive [1, 2]. An imperfect vaccine which provides partial protection could provide complete protection to some vaccinees and no protection to others (called “all-or-nothing” protection, or “take”-type protection), in which case its measured efficacy in a trial (or effectiveness in an observational study) is equivalent to the proportion of vaccinees getting complete protection [3]. Alternatively, an imperfect vaccine could provide partial protection to all vaccinees (called “leaky” protection, or “degree”-type protection), with the measured efficacy/effectiveness being equivalent to the average proportionate reduction in risk experienced by vaccinees [3] An imperfect vaccine will have a different population-level impact (ie, numbers of infections averted) when used at scale in a vaccination program, depending on whether it provides all-or-nothing protection versus leaky protection, with the former having greater impact [3].

Studies to date [1] have not determined whether 4CMenB's protection against gonorrhea is all-or-nothing or leaky. However, the findings of Raccagni et al [4] inform on this. An all-or-nothing vaccine will not modify the course of infection because vaccinees will either be fully protected (and hence not become infected) or have no protection (in which case if infection occurs then there will be no modification to the course of infection). In contrast, a leaky vaccine can have multiple effects [5]. Having previously reported that 4CMenB vaccinees had reduced rates of gonococcal infection, Raccagni et al's new analysis [4] indicates that the protection against *Neisseria gonorrhoeae* from 4CMenB is leaky because vaccination was associated with a reduction of almost half (from 66.7% to 34.2%) in the probability that an infection is symptomatic (ie, a reduction in gonorrhea disease per gonococcal infection). This is an important validation of a key assumption (ie, that protection is leaky) made in papers modeling the population-level impact of using an imperfect gonorrhea vaccine [eg 6–9], including the cost-effectiveness analysis [7], which underpinned the advice of the UK's Joint Committee on Vaccination and Immunisation that a vaccination program using 4CMenB be implemented [1].

Whilst the reduction in symptoms identified by Raccagni et al [4] is likely beneficial to patients overall, on average it would tend to prolong infection because symptoms typically prompt care-seeking and treatment within days, whereas even in groups being screened every 3 months the average time from asymptomatic infection to screening would be 1.5 months. A small but important proportion of infections result in sequelae such as disseminated gonococcal infection and pelvic inflammatory disease (in women, although we recognize that Raccagni et al's study was of men), and a prolongation of infection could increase this risk—although data to inform on this possibility are lacking at present. However, we note the striking increase (almost a trebling) in the proportion of infections that failed to produce a positive culture (from 19.6% to 55.3%) [4], which suggests that the bacterial load might have been reduced—which might offset the effect of prolonged infection regarding the risk of sequelae. There might also be a reduction in infectivity, reducing future numbers of infections, and need for treatment and thus helping reduce antimicrobial resistance risk—although we do not know the relationships between bacterial load, probability of infection upon exposure, and probability of culture-positivity.

An important implication is that where diagnostic algorithms (which might vary by jurisdiction, and by population group within a jurisdiction) specify that positive gonorrhea nucleic acid amplification test (NAAT) results be culture-confirmed for diagnosis and treatment, Raccagni et al's [4] findings suggest that the vaccination status of patients needs to be considered. It might be the case that the cycle threshold value of the NAAT result needs to be considered when interpreting NAAT-positive/culture-negative results. We recommend that vaccine studies report NAAT cycle threshold values, and that where possible studies perform culture testing of NAAT-positive samples—and ideally also of NAAT-negative samples—so that patterns of concordance in NAAT and culture can be understood, for individuals in both vaccine and control arms of studies.

As we have previously highlighted [5, 10], gonorrhea's natural history is poorly characterized, and vaccination studies can provide valuable data on the natural history of infection by examining individuals in the unvaccinated comparator group. One of the things we need to understand better is the proportions of infections at different anatomical sites that cause symptoms. Raccagni et al [4] report the proportion of diagnosed rectal infections that is symptomatic to be 34/51 (66.7% [95% confidence interval, 52.1–79.2]). We encourage others with suitable data to perform similar analyses as we need more studies, in different population groups (of women and men) and examining different anatomical sites [5, 10] (including the pharynx, which might play an important role in gonorrhea transmission and where antibiotic treatment might be less effective). We also need better estimates of rates of development of sequelae such as disseminated gonococcal infection and pelvic inflammatory disease, and the effects of vaccination on these rates—for which large population-based studies can be particularly useful. There is now a substantial number of gonorrhea vaccination studies [1] completed, ongoing, and planned, providing a golden opportunity to better understand gonorrhea's natural history at minimal cost, using data already collected or going to be collected. We are pleased that our letter [10] informed Raccagni et al's analysis [4]; we commend them on their valuable contribution, and we hope other studies will undertake the analyses we advocate.

## Notes

***Disclaimer.*** The views expressed are those of the authors and not necessarily those of the UK Department of Health and Social Care; Foreign, Commonwealth and Development Office; European Union; UK Medical Research Council (MRC); National Institute for Health and Care Research (NIHR); UK Health Security Agency (UKHSA); or Wellcome Trust.

***Financial support.*** This work was supported by the UK MRC Centre for Global Infectious Disease Analysis (grant number MR/X020258/1); this award comes under the Global Health EDCTP3 Joint Undertaking. This work was also supported by the NIHR Health Protection Research Unit in Modelling and Health Economics, which is a partnership between the UKHSA, Imperial College London, and the London School of Hygiene & Tropical Medicine (grant code NIHR200908). D.N. is funded by an MRC PhD studentship. L.K.W. is funded by the Wellcome Trust (grant number 218669/Z/19/Z).

The funding source(s) had no involvement in study design, analysis and interpretation of data, writing of the report, or the decision to submit the article for publication.

For the purpose of open access, the authors are required by the funders to apply a Creative Commons Attribution (CC BY) license to any Author Accepted Manuscript version arising.

## References

[1] Ladhani SN, White PJ, Campbell H, et al Use of a meningococcal group B vaccine (4CMenB) in populations at high risk of gonorrhoea in the UK. Lancet Infect Dis 2024; 24:e576–83. 38521080 10.1016/S1473-3099(24)00031-8

[2] White PJ, Leng T, Nikitin D, Whittles LK. The potential role of the 4CMenB vaccine in combating gonorrhoea: the need for nuance in interpreting the DOXYVAC trial. Lancet Infect Dis 2024; 24:e543. 39032496 10.1016/S1473-3099(24)00452-3

[3] Garnett GP . The theoretical impact and cost-effectiveness of vaccines that protect against sexually transmitted infections and disease. Vaccine 2014; 32:1536–42. 24606635 10.1016/j.vaccine.2013.11.007

[4] Raccagni AR, Diotallevi S, Lolatto R, et al Breakthrough rectal *Neisseria gonorrhoeae* infections after meningococcal B vaccination: microbiological and clinical features. Open Forum Infect Dis 2024; 11:ofae562. 39498171 10.1093/ofid/ofae562 PMC11532644

[5] Grad YH, Goldstein E, Lipsitch M, White PJ. Improving control of antibiotic resistant gonorrhea by integrating research agendas across disciplines: key questions arising from mathematical modeling. J Infect Dis 2016; 213:883–90. 26518045 10.1093/infdis/jiv517 PMC4760416

[6] Whittles LK, White PJ, Didelot X. Assessment of the potential of vaccination to combat antibiotic resistance in gonorrhea: a modeling analysis to determine preferred product characteristics. Clin Infect Dis 2020; 71:1912–9. 31905399 10.1093/cid/ciz1241 PMC7643747

[7] Whittles LK, Didelot X, White PJ. Public health impact and cost-effectiveness of gonorrhoea vaccination: an integrated transmission-dynamic health-economic modelling analysis. Lancet Infect Dis 2022; 22:1030–41. 35427491 10.1016/S1473-3099(21)00744-1 PMC9217755

[8] Nikitin D, Whittles LK, Imai-Eaton JW, White PJ. Cost-effectiveness of 4CMenB vaccination against gonorrhea: importance of dosing schedule, vaccine sentiment, targeting strategy, and duration of protection. J Infect Dis 2025; 231:71–83. 38630583 10.1093/infdis/jiae123 PMC11793026

[9] Hui BB, Padeniya TN, Rebuli N, et al A gonococcal vaccine has the potential to rapidly reduce the incidence of *Neisseria gonorrhoeae* infection among urban men who have sex with men. J Infect Dis 2022; 225:983–93. 34894134 10.1093/infdis/jiab581 PMC8922007

[10] White PJ, Nikitin D, Whittles LK. We need estimates of gonorrhoea vaccine protection and symptomaticity by sex and anatomical site. Lancet Infect Dis 2022; 22:937. 10.1016/S1473-3099(22)00343-7 35752179

